# Pathophysiological Mechanisms and Nonpharmacological Interventions in Irritable Bowel Syndrome: Current Insights and Future Directions

**DOI:** 10.1155/jnme/4520019

**Published:** 2026-01-13

**Authors:** Stefanie L. Y. Cheung, Leanne C. Kenway

**Affiliations:** ^1^ School of Pharmacy and Medical Sciences, Griffith University, Nathan, Queensland, Australia, griffith.edu.au

## Abstract

Irritable bowel syndrome, diagnosed using the ROME IV diagnostic criteria, is one of the most common dysfunctional disorders of the gastrointestinal system with a high global prevalence. Although symptom presentation is diverse, symptoms primarily manifest as abdominal pain, bloating, and alterations to bowel habits, negatively impacting quality of life but without an associated increase in mortality risk. Disruptions to the gut–brain axis, the bidirectional communication system between the central nervous system and the enteric nervous system, are hypothesised to be at the core of irritable bowel syndrome. Dysfunction may also be associated with stress and anxiety, as well as dietary factors, among other aspects related to physical and social environment, genetic predisposition and medical history. Patients with irritable bowel syndrome have also demonstrated increased vulnerability to neurotransmitter imbalances, with abnormalities associated with changes in gastrointestinal motility, low‐grade inflammation and visceral pain. Moreover, chronic stress and anxiety may significantly exacerbate symptoms through the upregulation of cortisol secretion, disrupting the gut microbiome and elevating visceral sensitivity. While the gut microbiome maintains the integrity of the gut–brain axis and intestinal barrier, decreases in its diversity heighten susceptibility to intestinal inflammation. Although there is currently no known cure for irritable bowel syndrome, research supports stress management and behavioural therapies, a low fermentable oligosaccharides, disaccharides, monosaccharides and polyols (FODMAP) diet, and probiotic supplementation as key interventions to alleviate symptoms. Additionally, faecal microbiota transplantation emerges as a promising intervention that addresses some of the limitations in current interventions. This literature review explores the pathophysiological mechanisms relating to irritable bowel syndrome, with insight into current interventions and future directions to directly address the underlying factors driving symptomology.

## 1. Introduction

Irritable bowel syndrome (IBS) is one of the most prevalent chronic functional gastrointestinal (GI) disorders, afflicting 10%–15% of the global population. However, prevalence varies between regions and environments and is influenced by perinatal and adolescent risk factors [1–3]. IBS also exhibits a significant sex‐related discrepancy, with diagnoses almost doubled in females compared to males, potentially attributed to psychosocial and hormonal factors [4, 5]. Beyond its high prevalence, IBS may be both physically and emotionally debilitating, having negative implications on the quality of life (QoL).

The clinical presentation of IBS is remarkably varied, including abdominal pain, bloating, flatulence and alterations to bowel habits [6]. The ROME IV Diagnostic Criteria outlines the clinical guidelines for IBS diagnosis, requiring recurrent abdominal pain for a minimum of one day per week in the past 3 months with a combination of two or more criteria relating to changes in defecation, stool frequency or stool form [7]. IBS can be classified into four subtypes based on predominant Bristol stool types: IBS‐C (constipation‐predominant), IBS‐D (diarrhoea‐predominant), IBS‐M (mixed bowel habits) and IBS‐U (unclassified). IBS‐C and IBS‐D are diagnosed when more than 25% of bowel movements involve constipation or diarrhoea, respectively. IBS‐M is characterised by more than 25% of bowel movements involving both constipation and diarrhoea. IBS‐U is diagnosed when the stool habits of the other subtypes are infrequent or absent, but the ROME IV Diagnostic Criteria are still met [7].

Currently, the symptom‐based Rome IV criteria are the gold standard for diagnosis, as no single diagnostic biomarker exists. However, research has revealed differences in urine metabolomics, blood biomarkers, faecal markers and immunological profiles [8, 9]. While no specific diagnostic test is available, a combination of symptom assessment and emerging biomarkers may aid in diagnosing IBS.

The aetiology of IBS remains exceedingly complex and multifactorial, yet to be fully understood. Key contributors include the intricate interplay between the nervous systems and digestive system, as well as psychological influences, neurotransmitters, GI motility, visceral sensitivity, inflammation and alterations to the gut microbiota [10, 11]. Risk factors for IBS are diverse, including genetic predisposition, demographics, environment, stress and anxiety, as well as medical history [12–14].

Current research suggests that the pathophysiology of IBS is linked to neurobiological factors. Disruption of gut–brain axis (GBA) homoeostasis, the bidirectional signalling network between the central nervous system (CNS) and the enteric nervous system (ENS), has been theorised to be at the core of IBS pathophysiology [11, 13, 15–17]. The components are linked to neural, hormonal and immunological pathways. These connections relate to neurotransmitter imbalances, pain processing, disruptions to the gut microbiome and altered GI motility [11, 13, 17, 18]. Stress and anxiety emerge as one of the most prominent triggers of IBS, exacerbating visceral pain and inflammation through the hypothalamic–pituitary–adrenal (HPA) axis [15]. Diet is also proposed to be a key factor influencing the gut microbiome, neurotransmitter expression and GI motility [10, 11, 18].

Current therapeutic interventions for IBS are limited and often unsustainable, providing temporary alleviation. First‐line pharmacological interventions include antispasmodic agents to slow intestinal transit [19]. Tricyclic antidepressants and 5‐hydroxytryptamine (5‐HT) Type‐3 receptor antagonists may modulate neurotransmitter and gut motility and tone; however, adverse effects and unfavourable drug interactions should be considered [20, 21]. Nonpharmacological interventions include stress and anxiety management such as cognitive behavioural therapy (CBT), along with dietary modification, including the low fermentable oligosaccharides, disaccharides, monosaccharides and polyols (FODMAP) or ketogenic diet. However, due to the highly individualised nature of IBS, a single cure remains undiscovered.

While IBS is chronic with debilitating potential, its multifaceted pathophysiology remains ambiguous due to insufficient evidence and inconsistencies and potential biases across research trials. Stress management and dietary modification are accessible interventions but present limitations. Although targeting psychological factors provides benefits, it is constrained by time demands and indirectly addresses GI symptoms. Dietary modification directly targets GI symptoms, but may contribute to micronutrient deficiencies, disordered eating patterns and altered microbiomes [10, 22, 23]. Enhancing stress management and dietary strategies to support GBA homoeostasis may provide a more targeted and sustainable approach to symptom relief, warranting further research to enhance these interventions.

## 2. Objective and Research Questions

This review delves into the contemporary understanding of the pathogenesis of IBS, encompassing the neural pathways and neurotransmitters involved, how they contribute to visceral hypersensitivity and inflammation, as well as the gut microbiome, with exploration of stress and anxiety management and dietary modification as key interventions to target the specific underlying mechanisms of IBS. The overarching research question guiding this review is: How do current management strategies for IBS address the underlying pathophysiological factors, and what evidence exists regarding their effectiveness?

To address this question, the review considers three main themes:

1. Pathophysiological mechanisms: GBA, neurotransmitters, stress, anxiety, depression, visceral hypersensitivity and gut microbiome
2. Therapeutic approaches: efficacy and sustainability
3. Integrative potential: future research

## 3. Methods

**Figure 1:**
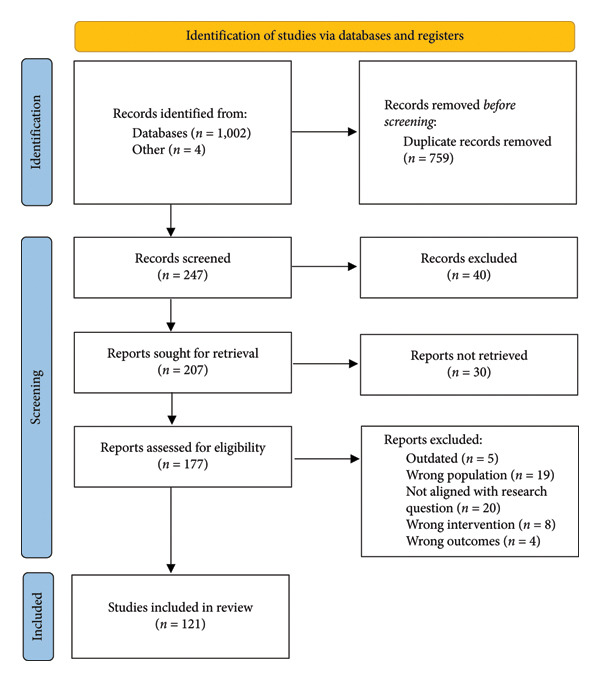
PRISMA flow diagram for the literature selection process.

Literature referenced in this review consists of a variety of peer‐reviewed articles, narrative literature reviews, systematic literature reviews and meta‐analyses, and clinical trials sourced through Griffith University Library Catalogue and Google Scholar, as well as PubMed, Embase and Wiley databases. Two primary search terms, ‘irritable bowel syndrome’ and ‘gut‐brain axis’, were used to gain insight into the broad context of IBS. A combination of secondary search terms, including ‘pathophysiology’, ‘neurobiology’, ‘visceral hypersensitivity’, ‘stress’, ‘diet’ and ‘gut microbiota’, were used to further narrow the literature for specific discussion sections. Literature in languages other than English was excluded. No formal date restriction was applied to the search; however, most included studies were published within the past 15 years, with several earlier references to provide context. The search included publications up to 2025, and grey literature and preprints were not systematically included. The abstract and conclusion of each literature piece were critically analysed to determine their relevance with respect to the research questions, and then, articles were organised into a Microsoft Excel spreadsheet. Figure 1 displays the PRISMA flow diagram, visually representing the number of records from identification to inclusion and reasons for exclusion [24].

## 4. Discussion

### 4.1. Bidirectional Communication Between the Brain and Gut

**Figure 2:**
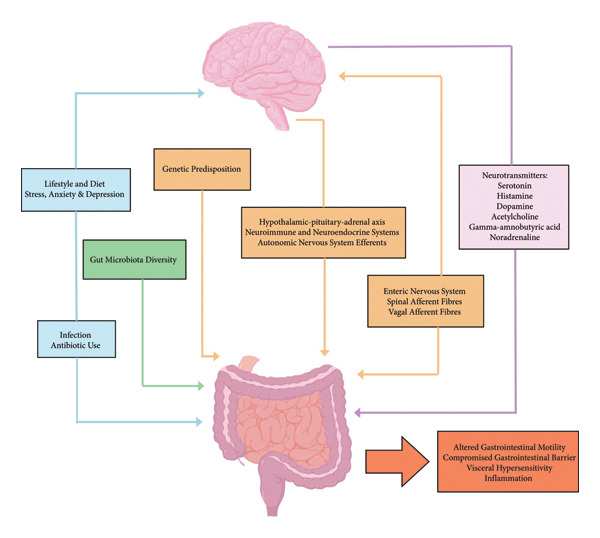
Interconnecting loops facilitate the bidirectional communication of the GBA.

IBS was first described as *mucous colitis* in the 19th century and believed to be either a psychosomatic or intestinal motility disorder [17, 25]. However, the first use of the current term IBS dates to the mid‐20th century, with modern research and scientific advancements now hypothesising it to be a GI disorder resulting from the dysbiosis and dysregulation of the GBA, often co‐occurring with psychiatric disorders [11, 13–15, 17, 18, 26–28]. The GBA is the bidirectional, reciprocal communication system between the CNS and the ENS [29]. In this connection, a reflex network of afferent fibres projects to the integrative cortical CNS structures, processing sensations in the gut, while a network of efferent fibres supplies the smooth muscle and glands in the intestinal wall, regulating motility and secretions [30]. This network plays a vital role in monitoring and integrating gut functions via the linkage of the emotional and cognitive brain centres with peripheral intestinal functions [29]. The interconnected components of the GBA encompass the CNS, autonomic nervous system (ANS), HPA axis, ENS, gut microbiome and neuroimmune and neuroendocrine systems [13, 27, 29], as presented in Figure 2.

The CNS and GI system communicate bidirectionally through various pathways of the GBA, including endocrine and immune signalling mechanisms. The GBA is interconnected through the CNS, ANS, HPA axis, ENS, gut microbiome and the neuroimmune and neuroendocrine systems. Genetics, environment, lifestyle factors including stress and diet, previous infections and antibiotic use also contribute to the complexity of the GBA.

The ANS, part of the efferent division of the peripheral nervous system, regulates involuntary body processes. It consists of two main branches: the sympathetic nervous system (SNS), responsible for the ‘fight, flight, or freeze’ response, and the parasympathetic nervous system (PSNS), governing ‘rest and digest’ activities. The ANS communicates bidirectionally with the CNS, receiving afferent sensory signals from the gut via enteric, spinal and vagal pathways and conducting efferent motor signals to regulate various organs and processes [16]. Acute stress activates the SNS, stimulating the adrenal medulla to release catecholamines, including adrenaline and noradrenaline, and concurrently suppresses the parasympathetic vagus nerve activity in the GI system. This stress‐induced activation of the SNS, with reduced PSNS activity, is hypothesised to play a significant role in IBS symptomology [31].

The vagus nerve, composed of 80% afferent fibres and 20% efferent fibres, is a key parasympathetic modulator of the GBA [12]. Typically, the vagus nerve is activated by diet‐responsive gut microbes and their metabolites to activate the neuromodulatory pathways and plays a critical role in transmitting information from the gut lumen environment to the CNS to drive the brain–body interactions [31, 32]. However, during periods of stress, vagal tone decreases, thus heightening vulnerability to intestinal inflammation [10, 33].

The HPA axis is the core stress efferent axis, and a key neuroendocrine mechanism regulated by the gut microbiota which influences its activity through metabolites. Prolonged activation of the HPA axis by chronic stress stimulates the excess release of cortisol, the primary stress hormone [29]. The HPA axis is regulated at the level of the hypothalamus through afferent projections from the limbic system, amygdala, mid‐brain and brain stem. While current research on HPA axis dysfunction shows inconsistent findings, many researchers agree that this dysfunction results in a mismatch between neural and hormonal pathways, contributing to symptoms of stress and anxiety. This mismatch is associated with increased intestinal barrier permeability and heightened visceral pain, also playing a role in the pathophysiology of GBA dysfunction, characterised by GI‐related symptoms and low‐grade inflammation [27, 34].

The ENS, often regarded as the ‘second brain’, is located within the walls of the gastrointestinal tract (GIT) and can operate independently of the CNS. It regulates the musculomotor, neurohormonal and secretory systems of the gut and is hypothesised to contribute to IBS symptomology [35]. The ENS plays a pivotal role in the GBA, connecting with the spinal cord, brainstem and brain through spinal and vagal afferent fibres, as well as sympathetic efferent fibres, allowing for the integration of viscerosensory signals [17, 36]. Abnormal GI motility and visceral hypersensitivity are often attributed to an imbalance of neurotransmitters produced by the ENS, including 5‐HT, dopamine, gamma‐aminobutyric acid (GABA) and histamine. These imbalances contribute to the dysfunction in the bidirectional communication network between the enteric microbiota and the brain [17, 18, 26, 29].

Current research strongly supports the hypothesis that GBA dysfunction is the core of IBS, with researchers demonstrating comprehensive knowledge of the individual aspects of the GBA. Research in this area is now being directed to develop targeted treatments for the specific connections.

### 4.2. Neurotransmitters in GI Motility and Low‐Grade Inflammation

While GBA dysfunction is hypothesised to be one of the greatest contributors to IBS symptoms, neurotransmitters also influence connections within the GBA, where imbalances contribute to visceral pain, altered GI motility and inflammation. Neurotransmitters are chemical messengers that transmit various signals between nerve cells to regulate diverse processes. The primary neurotransmitters identified in IBS pathophysiology include 5‐HT, histamine, dopamine, acetylcholine, GABA and noradrenaline, with their imbalances contributing to alterations in GI motility and low‐grade inflammation.

### 4.3. Serotonin

Serotonin, or 5‐HT, has received substantial attention in IBS research, where abnormal levels drive IBS symptoms. Over 90% of serotonin is synthesised and released from the enterochromaffin cells within the innermost mucosa lining the GIT, acting as a messenger for GI motility, visceral sensation and fluid secretion in the gut [18, 31, 37]. In IBS patients, alterations in serotonin reuptake transporter (SERT) transcription and expression, influenced by both genetic and environmental factors, disrupt serotonin regulation, contributing to the symptoms associated with different IBS subtypes [38]. The remaining serotonin is produced in the CNS, regulating cognition, mood and other physiological processes [28].

Bülbring and Crema [39] were among the first to associate 5‐HT with the peristaltic reflex, where stimulation of 5‐HT secretion from the gut promotes peristalsis, providing further insight into the pathogenesis of IBS. Stimulation of 5‐HT3 receptors increases sensitivity in the ENS and visceral afferent nerves, stimulating motor and secretory reflexes associated with symptoms such as nausea, vomiting, discomfort, abdominal pain and inflammation, particularly in IBS‐D [26, 40, 41]. 5‐HT4 receptor activation stimulates internal sensory neurons, regulating GI motility via smooth muscle modulation, and agonists may alleviate symptoms of IBS‐C [42, 43].

Cremon et al. [44] found serotonin levels in the colon to be 10 times greater in IBS patients compared to controls. Similarly, in a study by Chojnacki et al. [45] involving 32 clinically healthy participants, 40 IBS‐D and 36 IBS‐C participants diagnosed using the Rome IV criteria, serotonin levels were highest in IBS‐D participants (212.1 ng/mL), followed by clinically healthy participants (201.2 ng/mL), and the lowest in IBS‐C participants (140.9 ng/mL). Despite variability across studies, targeting specific serotonin receptors holds potential for drug development—slowing or enhancing gut motility to alleviate subtype‐specific IBS symptoms.

### 4.4. Histamine

Histamine is a proinflammatory mediator released during mast cell degranulation, increasing vascular permeability, stimulating smooth muscle contraction and triggering sensory nerves. Its role in IBS is hypothesised to be related to GI motility and low‐grade inflammation, intestinal permeability, increased secretions and pain via stimulation of visceral sensory nerves [26]. Barbara et al. [46] found that when mucosal biopsies from IBS patients were incubated in oxygenated buffer (95% O_2_/5% CO_2_) at 37°C, significant quantities of histamine were released within 5 minutes (146.7 ± 28.5 ng/mL/mg). Histamine release further increased after 25 min (182.1 ± 27.2 ng/mL/mg), with increases of 118% and 354%, respectively, compared to healthy controls. Smolinska et al. [47] also confirmed elevated histamine levels in IBS patients through colonic mucosal biopsy samples, supporting findings of increased histamine release in this population. The increased histamine stimulates neuronal activity in addition to upregulating H1 receptors and is associated with proinflammatory effects and visceral pain [26, 46, 48]. Although the influence of histamine has been studied extensively in animal models, more research is necessary to understand its full effects in humans and, in particular, IBS patients. Histamine receptor antagonists, such as antihistamines, may be further explored as potential pharmaceutical interventions to block the downstream effects of histamine and alleviate visceral pain in IBS patients.

While research observing the effect of neurotransmitters in IBS still presents inconsistent results, there is strong evidence linking neurotransmitter imbalances to visceral pain, altered GI motility and low‐grade inflammation. This understanding underscores the potential for pharmaceutical interventions to restore neurotransmitter balance, offering promising therapeutic interventions to manage IBS symptoms more effectively.

### 4.5. Stress, Anxiety and Depression in IBS Symptoms

Due to the numerous overlapping GI and psychological factors involved in IBS pathophysiology, controversy has surrounded whether IBS should be considered a GI or a psychiatric disorder. Substantial evidence now supports the perspective that IBS is a functional GI disorder resulting from GBA dysfunction, which contributes to GI symptoms via a compromised intestinal barrier and increased visceral sensitivity [23, 26, 49]. Despite this, stress and anxiety remain significant triggers for symptom flare‐ups in many patients.

In a multivariate analysis involving 769 participants, 44.9% of IBS participants reported having anxiety and 25.7% depression based on the Hospital Anxiety and Depression Scale, where participants reporting psychological distress experienced greater GI‐specific anxiety based on the Visceral Sensitivity Index. Additionally, the IBS‐QoL Questionnaire, comprised of nine scales, found that IBS patients with psychological distress had an overall lower QoL [50]. In an earlier study in India with 35 IBS patients and 35 healthy controls, 31.4% of IBS patients reported anxiety and 37.1% depression based on the Hamilton Anxiety Rating Scale and Hamilton Depression Rating Scale, respectively [51]. Although the prevalence of anxiety and depression in IBS patients varies across studies due to differences in environments and regions, selection criteria and potential sampling biases, the overall results consistently demonstrate a higher prevalence of anxiety and depression in IBS patients compared to the general population.

During periods of stress, both the HPA axis and the SNS initiate physiological responses through the secretion of glucocorticoids and catecholamines. In the short‐term stress response mediated by the SNS, catecholamines, including adrenaline and noradrenaline, are secreted from the adrenal medulla, leading to increased heart rate, blood pressure and metabolic rate. In contrast, the chronic stress response, regulated by the HPA axis, involves the secretion of corticotropin‐releasing hormone (CRH) from the hypothalamus into the hypophyseal portal circulation. This stimulates the release of adrenocorticotropic hormone (ACTH) from the anterior pituitary gland, which in turn promotes cortisol release from the adrenal cortex. Cortisol raises blood glucose levels and blood pressure while exerting immunosuppressive effects [52].

Elevated cortisol levels are associated with IBS patients, who show substantially higher baseline levels (430.2 ± 20.6 nmol/L) compared to healthy controls (329.1 ± 14.4 nmol/L). Additionally, IBS patients exhibit greater CRH and ACTH responses [53]. In another study, under prolonged stress, the cortisol/dehydroepiandrosterone ratio was disproportionally higher in IBS patients compared to healthy controls, most notably 30 min after awakening, and cortisol effects exacerbated IBS symptoms [54]. In an older study, the results showed a substantial increase in serum cortisol levels in 10 IBS patients compared to 10 healthy controls despite identical CRH levels (from baseline to peak at 60 min, controls: 13.6 (1.2) mg/dL to 28.9 (1.3) mg/dL; IBS patients: 13.6 (1.6) mg/dL to 30.2 (1.8) mg/dL; *p* < 0.001) [55]. Although all three studies selected participants with IBS using established diagnostic criteria, discrepancies in the extent of cortisol elevation may potentially be attributed to differences in study design, sample size or potential random sampling error when selecting participants, as the studies had 151 (76 IBS patients and 75 controls), 33, and 20 (10 IBS patients and 10 controls) participants, respectively [53–55].

While elevated cortisol levels contribute to visceral hypersensitivity, stress and anxiety in individuals with IBS have also been linked to low‐grade inflammation and disruptions to the GBA and gut microbiome. Considering the high prevalence of anxiety and depression in IBS, mental health disorders and other psychological factors may be targeted for developing future therapeutic interventions. Addressing these aspects may allow for the effective alleviation of both GI‐ and non–GI‐related symptoms.

### 4.6. Visceral Hypersensitivity in IBS

**Figure 3:**
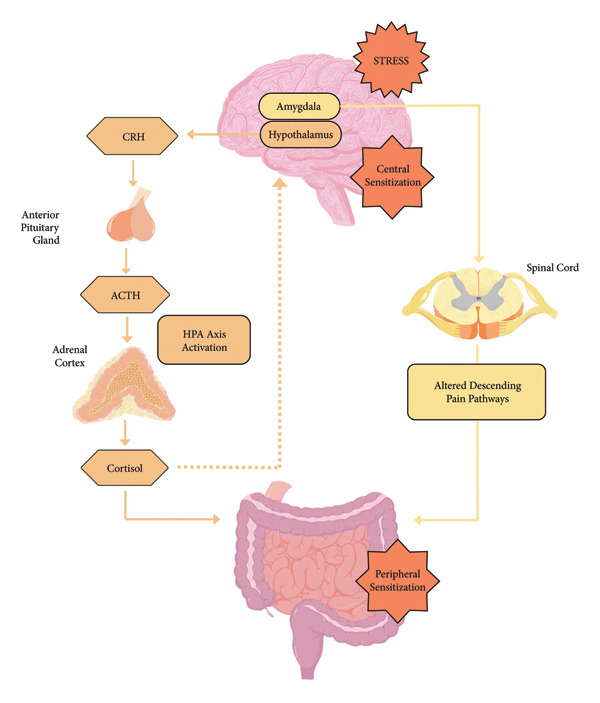
Central sensitisation and peripheral sensitisation in visceral hypersensitivity.

Visceral pain, originating from the internal organs, is often poorly localised, and current interventions and management strategies targeting the pain are generally ineffective and unsustainable. Visceral hypersensitivity has a key role in abdominal nociception, with involvement of central and peripheral mechanisms, and may be detected in up to 60% of IBS patients [46]. Current evidence suggests that stress and anxiety play a central role in visceral sensitivity, contributing to GBA alterations, intensifying signals from the ENS to the CNS, activating the HPA axis and increasing susceptibility to low‐grade intestinal inflammation. Together, these factors heighten sensitivity to GI pain in IBS [56], as depicted in Figure 3. These factors influence visceral nociception by sensitising pain pathways, consequently increasing visceral pain [15, 57]. Additionally, stress coupled with chronic, low‐grade inflammation of the intestinal mucosa may drive visceral hypersensitivity in the lower GIT [58].

Visceral hypersensitivity is measured in response to distension in specific regions of the GIT, where a balloon catheter is inserted into the colorectal region with graded isovolumetric distensions, and perception of pain and discomfort are then matched with thresholds [59]. Multiple studies have consistently found that IBS patients report lower pain thresholds in response to visceral stimuli compared to those without IBS. A study found that hypersensitive IBS patients, when exposed to high‐ and low‐intensity rectal distension, demonstrated regional reductions in the brain blood oxygen level–dependent (BOLD) response in brain regions related to pain sensation. In hypersensitive IBS patients, high‐intensity distension decreased BOLD activity in regions such as the right dorsal insula, right ventral insula and left posterior insula, as well as parts of the prefrontal cortex. For low‐intensity distension, BOLD reductions occurred in areas including the amygdala, left and right dorsal anterior insula and the left posterior insula. In contrast, IBS patients without visceral hypersensitivity exhibited no significant reductions in the BOLD response during repeated distensions under similar conditions [60]. In hypersensitive IBS patients, high‐intensity colorectal distension was associated with altered brain activation patterns, including reduced deactivation in the posterior anterior cingulate cortex (ACC) and changes in the activation of the pregenual ACC, the amygdala and other brain areas involved in pain processing, reflecting disruptions in the engagement of descending pain modulation systems [61]. While several studies have highlighted significant differences in ACC activation between those with and without IBS, the extent of activation was inconsistent among studies, potentially due to the methods used.

Stress stimulates HPA‐axis activation, resulting in increased cortisol secretion and heightened peripheral sensitisation. Sensitised spinal afferents manifest as lower pain thresholds to distension. Alterations to pain processing also contribute to heightened visceral sensitivity.

### 4.7. Low‐Grade Inflammation in IBS

Low‐grade inflammation in the mucosa has been linked to IBS, where increased intestinal permeability enables the abnormal gut microbiota to drive inflammation, elevate circulating cytokines and increase visceral hypersensitivity [13, 62]. In a study of 180 participants (90 IBS patients and 90 controls), IBS patients showed elevated proinflammatory cytokines, including tumour necrosis factor‐α (TNF‐α), which increases vascular permeability, and interferon gamma, associated with immune system dysregulation. Levels of interleukin (IL)‐17 were higher and IL‐10 levels lower, particularly in the IBS‐D subtype. Specifically, serum TNF‐α was significantly elevated in IBS‐D patients compared to controls (21.7 ± 3.96 vs. 10.04 ± 5.75 pg/mL, *p* < 0.001), as were IL‐17 levels (12.76 ± 2.8 vs. 5.63 ± 3.15 pg/mL, *p* < 0.001), while IL‐10 was lower (5.65 ± 1.62 vs. 9 ± 3.23 pg/mL, *p* < 0.001). Serum levels of these cytokines were similar in IBS‐C and IBS‐M patients [63]. A meta‐analysis by Bashashati et al. [64] confirmed elevated TNF‐α and IL‐6 levels in IBS‐D, which influence inflammation through heat and energy metabolism. Dinan et al. [53] found similar increases in IL‐6 across all IBS subtypes, with higher IL‐8 levels in IBS‐C. Mitselou et al. [65] emphasised the role of TNF‐α in IBS pathogenesis, noting higher mucosal levels in IBS patients compared to irritable bowel disease (IBD) patients and healthy controls. Bennet et al. [66] also found increased IL‐6 and IL‐8, and decreased IL‐10 in IBS. Overall, these findings suggest that dysregulated immune responses and altered cytokine profiles, particularly in IBS‐D, play a significant role in the pathophysiology of IBS, highlighting the potential for targeting inflammation and immune modulation in therapeutic interventions.

Chronic stress, dietary habits, genetic predisposition, prior infections, inflammation and alterations to the gut microbiota have been identified as contributors to increased intestinal permeability [16, 56, 57, 67]. This disruption, also characterised as a ‘leaky gut’, contributes to low‐grade intestinal inflammation. Intestinal permeability is measured by the level of expression of tight junction proteins and is increased by proinflammatory cytokines, while cytokine antagonists may offer a protective effect against inflammation [34, 62]. Although the gut microbiome exists in symbiosis with the GIT, a shift in microbiota composition may be a pathogenic factor if microbiota is displaced from the gut lumen into the ENS, immune cells or systemic circulation via a compromised intestinal barrier, potentially manifesting as low‐grade inflammation [12, 13]. Thus, anti‐inflammatory cytokines may provide therapeutic benefits against inflammation stemming from a leaky gut, and cytokine expression can be influenced by diet and microbiota composition, highlighting the complex interplay between these factors.

Toll‐like receptors (TLRs), present on immune cells, enteroendocrine cells and neurons of the ENS, play a crucial role in regulating inflammatory pathways and immune responses in the GIT. Additionally, TLRs may enhance the intestinal barrier by reinforcing tight junctions [16]. TLR expression is elevated in IBS patients, and increased TLR expression and immune activation are associated with alterations in gut microbiota profiles and elevated levels of proinflammatory cytokines, contributing to hypersensitivity and prolonged gut inflammation [12, 68]. However, the mechanisms underlying the increase and overstimulation of TLRs still remain uncertain.

Low‐grade inflammation in IBS arises from altered mast cell activation, abnormal cytokine levels and TLR expression, increased intestinal permeability and dysbiosis. Psychological stress and GBA dysfunction further exacerbate these effects, bridging immune activity with symptom severity and perception. These findings identify inflammation as a key mechanism in IBS pathophysiology, highlighting the therapeutic potential to target both immune modulation and stress to alleviate symptoms.

### 4.8. Alterations to the Gut Microbiome

The gut microbiome consists of the trillions of symbiotic microorganisms of bacterial origin within the GIT, covering over 200 m^2^ of mucosa, and is involved in immunity, maintenance of the intestinal barrier, maturation and development of the GIT and immune system, and facilitates the bidirectional communication of the GBA [13, 15, 16, 26, 29].

Colonisation of the gut by microbiota in early life is also hypothesised to establish the developmental conditions for HPA‐axis maturation, where early‐life stress results in lifelong alterations in microbial composition [69, 70]. Several animal studies have linked early‐life stress to long‐term changes in microbial composition, increasing the risk of developing IBS later in life. The ability to overcome the effects of stressors ultimately depends on the capacity of an individual’s HPA axis to adapt [27, 49].

The colonising microbiota, determined by environmental factors, is vital to the early development of the gut microbiome; however, there is an optimal period for colonisation to ensure the healthy development of the GBA [70, 71]. Early‐life risk factors for IBS include shorter breastfeeding duration (5.6 months vs. 8.1 months, *p* = 0.009) and caesarean delivery, which results in colonisation by maternal epidermal flora rather than vaginal flora. These factors may impact immune system development and gut health by delaying or limiting the colonisation of beneficial bacteria, ultimately altering gut microbiota composition and influencing GI health later in life [72, 73]. The role of low birthweight as a risk factor for IBS remains uncertain; it is unclear whether low birthweight itself predisposes individuals to IBS or if it reflects underlying developmental issues that increase the risk of IBS development [2].

Early life adversity (ELA) includes traumatic experiences during childhood, such as physical, sexual and emotional abuse, as well as general trauma. A study by Park et al. [74] involving 148 IBS patients found a strong correlation between ACE scores and Early Trauma Inventory (ETI) scores, which measure ELA. The study highlighted that ELA significantly increased the odds of developing IBS, where key predictors included household mental illness, emotional abuse and the presence of an incarcerated household member. Similarly, a study by Videlock et al. [75] with 44 IBS patients and 39 healthy controls found that 21 IBS patients and 18 controls had a history of ELA. In the study, patients with ELA exhibited a heightened cortisol response to visceral stressors compared to controls, with higher mean cortisol levels (0.32 ± 0.2 vs. 0.1 μg/dL; *p* = 0.003), a rapid cortisol peak and slower return to baseline. These physiological changes were associated with symptom exacerbation and reduced QoL. In a study of 344 participants aged 3–18, including 115 with ELA and 229 controls, a positive association was observed between ELA and GI symptoms. The ELA group exhibited altered microbiome diversity, including changes in alpha (richness) and beta (uniqueness) diversity [76]. However, a cross‐sectional study by Coley et al. [77] with 128 participants who completed the ETI found no significant relationship between ELA exposure (mean score 8.6 vs. 1.2) and microbial alpha diversity.

The hygiene hypothesis, proposed by epidemiologist Dr. David Strachan in 1989, suggests that reduced early‐life exposure to infectious agents due to improved sanitation and hygiene practices and environments may result in an underdeveloped immune system. This lack of microbial exposure could lead to an inappropriate immune response later in life, contributing to abnormal inflammatory processes [72, 78, 79]. While the hypothesis is well‐supported in explaining the rise of immune‐related conditions like allergies, asthma and autoimmune disorders, its applicability to the onset of IBS remains limited compared to its connection with IBD. More extensive research is needed to clarify the role of the hygiene hypothesis in IBS development.

Current research links psychological, dietary and lifestyle factors, prior infections, antibiotic use and genetic predisposition to alterations in the gut microbiome and intestinal permeability. These changes often include a significant loss of microbial diversity, a hallmark of dysbiosis consistently supported by studies and are associated with neurological disorders and autoimmune diseases. Although relatively stable, the gut microbiota continuously evolves over time, losing diversity and shifting towards a proinflammatory profile in response to environmental and physiological factors, including lifestyle changes and ageing [80, 81]. This loss of diversity, or dysbiosis, may drive inflammation, oxidative stress and increased intestinal permeability, contributing to visceral hypersensitivity and other symptoms [82, 83]. It also contributes to disruption of the GBA, influencing blood–brain barrier permeability and promoting neuroinflammation [84]. However, it remains unclear whether dysbiosis precedes GBA dysfunction or vice versa. Additionally, while studies consistently identify differences in the gut microbiota between IBS patients and healthy controls, no signature microbiome profile has been established to identify IBS patients. Accounting for the multitude of factors that disrupt gut microbiota composition in IBS patients, dietary modification may be a suitable microbiome‐targeted intervention. Long‐term dietary modification may involve including or excluding specific foods or supplementation with probiotics to restore balance within the gut microbiome and effectively manage symptoms.

### 4.9. Synergistic Mechanisms in IBS Pathophysiology

IBS is highly complex and multifactorial, involving synergistic interactions among the GBA, neurotransmitter imbalances, heightened cortisol, visceral hypersensitivity, inflammation and alterations in the gut microbiota. Microbial metabolites and neurotransmitters such as serotonin and histamine influence ENS function, immune modulation and HPA‐axis regulation, thereby affecting gut motility and visceral sensation [13, 15, 26, 29, 82]. Dysbiosis and low‐grade inflammation compromise the intestinal barrier, increasing permeability and triggering immune activation with cytokine release, which sensitises neural pathways and contributes to visceral hypersensitivity. Heightened stress and HPA‐axis activation elevate cortisol levels and disrupt autonomic regulation, further compromising barrier integrity and microbiota composition [27, 49].

The GBA maintains GI homoeostasis via bidirectional communication between the nervous system and intestinal microbiota. Dysregulation of this axis drives visceral hypersensitivity, abnormal motility and heightened stress responses. Dysbiosis also disrupts mucosal integrity, alters epithelial permeability and disrupts immune and neural signalling [85]. Microbial metabolites, including short‐chain fatty acids and tryptophan derivatives, and neurotransmitter precursors modulate ENS function and influence CNS activity indirectly via vagal afferents or immune mediators, as many microbial neurotransmitters are unable to cross the blood–brain barrier. CNS‐derived stress responses may also disrupt gut permeability and microbial composition, establishing a feedback loop. Additionally, nutrient bioavailability, modulated by the microbiota, impacts endocrine function and neurohormonal signalling [85, 86].

The microbiota also plays a crucial role in regulating intestinal barrier function, immune activation and sensory afferent pathways. Bacterial metabolites stimulate mucosal serotonin release, affecting gut motility and ENS signalling. Dysbiosis may activate mucosal immune responses, increasing permeability and inflammation to further exacerbate visceral pain and IBS symptoms [29]. These findings demonstrate that gut microbiota imbalances and GBA dysfunction interact synergistically in IBS, amplifying symptom severity and driving dysregulation. This highlights the potential for therapeutic strategies targeting both the gut microbiome and neuro‐enteric pathways to alleviate IBS.

## 5. Nonpharmacological IBS Interventions

### 5.1. Stress and Anxiety Management

Stress and anxiety have been associated with exacerbation of IBS symptoms, including visceral hypersensitivity and low‐grade inflammation, acting through elevated cortisol levels to first disrupt the GBA and gut microbiome. Traditional stress and anxiety management techniques for IBS include mindfulness and meditation, while other formal frontline therapeutic interventions for IBS involve counselling or CBT. CBT was first developed in the 1960s by Dr. Aaron T. Beck and is a vital subset of psychotherapy utilising an array of treatment modalities targeting cognition and behaviours driving distorted thinking patterns and ‘automatic thoughts’ [87]. The objectives of CBT are to transform one’s perspective on IBS into hopefulness; recognise the connection between cognition, feelings and behaviours relating to the environment and symptoms; and identify and employ coping mechanisms to improve QoL [54, 88]. In one study evaluating the impact of CBT on IBS, 69% of participants met the criteria for a positive clinical response on the IBS Symptom Severity Scale [89]. A positive response is defined as a reduction of over 50 points on the IBS Symptom Severity Scale [90]. Responders reported significant improvements in abdominal pain, overall symptom intensity, perceived stress and positive mood ratings. Microbiota analysis revealed that CBT responders at baseline had a distinct microbial shift, with increased *Clostridiales* and decreased *Bacteroidales*, but trended towards a *Bacteroides*‐dominant microbiota by the end of treatment. At baseline, neurotransmitter measurements also indicated that responders exhibited higher faecal serotonin levels, though dopamine and histamine levels remained similar between groups [89].

While CBT offers promising potential, differences in IBS subtypes and psychological profiles, the self‐reported nature of symptom improvement, as well as limited long‐term data, remain key limitations in determining its efficacy and sustainability. Although microbial shifts may be observed, it also remains uncertain whether the therapy directly improves symptoms or if these changes occur secondary to stress reduction. However, these findings suggest that CBT may alleviate IBS symptoms through modulation of brain–gut–microbiome interactions, highlighting the potential for psychological interventions to influence gut microbiota and neurotransmitter secretion in IBS pathophysiology.

Gut‐directed hypnotherapy has emerged as a promising psychological intervention aimed at inducing a state of deep relaxation, although its effectiveness remains uncertain due to variability in study results and differences in study design. The goal of gut‐directed hypnotherapy is to enable patients to gain greater control over their gut function, potentially improving GI function and overall well‐being. While the exact mechanisms underlying its effects are yet to be fully confirmed, studies have demonstrated positive impacts on gut–brain interactions, GI function and physiology [91]. In a study involving 150 IBS patients, 121 (81%) reported at least a 50‐point reduction in the IBS Symptom Severity Scale, with substantial improvements in pain severity, pain frequency, abdominal bloating severity, bowel habit satisfaction and overall life interference. Furthermore, this study reported an increase in QoL scores from a mean of 238.4 pre‐hypnotherapy to 306.7 post‐hypnotherapy (maximum score = 500), as well as notable reductions in anxiety and depression scores (pre‐hypnotherapy: 12.1 and 8.0, post‐hypnotherapy: 8.9 and 5.3, respectively) based on the Hospital Anxiety and Depression Scale [92]. Another study with 1000 participants revealed that 760 patients (76%) achieved a 50‐point or more reduction in symptom severity, with the mean QoL score rising from 264.6 pre‐hypnotherapy to 330.8 post‐hypnotherapy. There were also significant reductions in anxiety and depression scores (from 11.1 to 7.0 pre‐hypnotherapy to 8.0 and 4.6 post‐hypnotherapy, respectively) according to the same scale [93]. These findings suggest that gut‐directed hypnotherapy has the potential to be an effective treatment for IBS, contributing positively to symptom relief and QoL. However, study designs vary in duration and protocol standardisation, and the mechanism of hypnotherapy also remains uncertain. Long‐term data are also currently limited; hence, the degree of efficacy is variable. While hypnotherapy is gaining more recognition as a nonpharmacological intervention for IBS, further research is required to better understand its mechanisms and broaden its clinical application.

By targeting psychological factors, IBS patients may indirectly experience symptom alleviation; however, the evidence and magnitude of their effects still require extensive research to address GI‐related symptoms directly.

### 5.2. Dietary Modification

The rise of globalisation and urbanisation has led to greater adoption of the Western diet, characterised by processed foods high in sugars, sodium and unhealthy fats, and low in fibre and whole grains. In a study by Buscail et al. [94] involving 2423 IBS participants, the Western diet was associated with a moderate increase in IBS risk (OR Q5 vs. *Q*1 = 1.38, 95% CI 1.19–1.61, *p* < 0.0001). Another study found that 89.6% of 135 IBS patients identified diet as a trigger of their symptoms, with 91.9% needing to limit or exclude specific foods to prevent an exacerbation [95]. A prospective cross‐sectional observational study by Melchior et al. [96], with IBS patients from the French Association of IBS, found that 79.8% of patients experienced food as a trigger of symptoms in 38.6% of meals. In a different study by Melchior et al. [97] with 995 IBS patients, 13.2% practised severe food avoidance to manage symptoms. While findings vary due to sample size and biases, diet was consistently found to exacerbate IBS symptoms, likely disrupting gut microbiota. Low FODMAP, Mediterranean, DASH and ketogenic diets are dietary interventions often experimented with by IBS patients to prevent GI symptoms.

Despite diet being recognised as a key trigger of symptoms, individual responses are highly variable, attributed to differences in gut microbiota composition, visceral sensitivity and psychological factors. Strict food avoidance poses risks of nutritional deficiencies and poor long‐term adherence. Additionally, the self‐reporting of symptom changes, along with a lack of objective biomarkers, adds to the complexity of assessing the efficacy of dietary modifications. Hence, personalisation is recommended to optimise nutritional adequacy and promote long‐term adherence.

### 5.3. Low FODMAP Diet and Ketogenic Diet for IBS

FODMAPs are a group of short‐chain fermentable carbohydrates, which, when consumed and fermented, enhance the production of short‐chain fatty acids and gases contributing to luminal distension, bloating and abdominal discomfort [6, 98]. High FODMAP foods include fructose, found in honey and fruits like mangoes and figs; lactose, present in dairy products; fructans, found in foods like wheat, broccoli and onions; galactans, present in legumes; and polyols, such as sugar alcohols and certain fruits with pits or seeds [99–101]. Conversely, low FODMAP foods include nonpitted, nonseeded fruits low in fructose, such as bananas and oranges; dairy‐free alternatives; grains like oats and rice; nuts and seeds; and vegetables such as bean sprouts and carrots [99]. When FODMAPS are digested, IBS patients show increased sensitivity to the breakdown of the short‐chain carbohydrates, resulting in a greater osmotic effect in the gut lumen, as water is drawn into the small intestine to cause luminal distension and visceral pain [102]. IBS patients also exhibit heightened sensitivity to the fermentation process by which gut bacteria break down undigested food particles in the colon. This leads to increased production of short‐chain fatty acids and gases such as hydrogen, methane and carbon dioxide, contributing to flatulence, bloating and other GI symptoms [101, 103, 104]. In a different study with 82 IBS participants—43 participants following a low FODMAP diet and 39 following standard dietary advice—up to 86% of IBS participants on the low FODMAP diet reported significant improvement in symptoms, compared to 49% of those following standard dietary advice [105].

While strong evidence across numerous studies supports a low FODMAP diet as the primary dietary intervention, few studies address potential risks from long‐term adherence. Such risks include alterations to the gut microbiome, as well as vitamin, mineral and micronutrient deficiencies [103]. Even with the gradual reintroduction of high FODMAP foods, depriving the body of carbohydrates and prebiotic substrates may potentially promote the fermentation of proteins and amino acids to yield toxic by‐products, such as amines and sulphurous compounds [106, 107].

Similar to the low FODMAP diet, the ketogenic diet strives to minimise fermentation via the near elimination of carbohydrates. However, the ketogenic diet is high in fats and protein but low in fibre. Although posing controversy, researchers have found that the ketogenic diet may offer neuroprotective effects and modulate the gut microbiota and neurotransmitter secretion to restore the GBA [28]. Without carbohydrates as the body’s primary energy source, the body will induce a state of ketosis to burn ketones. Ketosis is thought to enhance cognition, and a randomised control trial by Iacovides et al. [108] found that ketosis had no impact on mood or sleep quality compared to individuals on a regular balanced diet. Ultimately, further research is needed to address the viability of ketogenic as a long‐term intervention for IBS, including weighing its potential benefits against associated risks, such as nutrient deficiencies, elevated low‐density lipoprotein cholesterol levels and increased risk of cardiovascular disease.

While both the low FODMAP and keto diet minimise fermentation in the gut through carbohydrate restriction to alleviate flatulence, bloating and other GI‐related symptoms of IBS, long‐term adherence to these highly restrictive diets is unsustainable and may pose potential health risks. Therefore, further research is needed to develop a more balanced dietary approach that alleviates IBS symptoms while maintaining gut integrity and overall nutritional health.

### 5.4. Probiotics to Restore the Gut Microbiome

As alterations and reduction of gut microbiota diversity are associated with a cascade of GI‐ and non–GI‐related disturbances in IBS, researchers have proposed probiotic supplementation as an intervention to restore the health of the gut microbiome.

The World Health Organization described the scope of probiotics as ‘live microorganisms which, when consumed in adequate amounts as part of food, confer a health benefit on the host’ [109]. Probiotics may play a role in modulating the gut microbiota, intestinal barrier, visceral hypersensitivity and GI motility [23]. Administration of probiotics may restore the functionality of the gut microbiota by replacing missing microorganisms, stimulating existing microorganisms or supplementing the endogenous population [110]. Raskov et al. [16] found that over 80 trials involving 10,000 patients have associated probiotic supplementation with the alleviation of symptoms, such as bloating and irregular bowel habits, with minimal to no adverse effects.

A meta‐analysis by Xie et al. [111], consisting of 9253 IBS participants across 81 randomised control trials, found *Lactobacillus acidophilus DDS-1* to be among the most effective probiotics compared to placebos in improving the IBS Symptom Severity Scale. A mixture of probiotics comprised of *Lactobacillus plantarum, Lactobacillus casei*, *Bifidobacterium longum, Saccharomyces boulardii, Lactobacillus acidophilus* and an unspecified probiotic was also effective in improving the IBS‐QoL scale. *Clostridium butyricum CGMCC0313.1* was superior to the placebo in improving QoL. *Bacillus coagulans MTCC 5856* and *Bacillus coagulans Unique IS2* emerged among the most effective probiotics for alleviating abdominal pain, while the top three treatments for abdominal bloating included *Bacillus coagulans MTCC 5856, Lactobacillus plantarum* and a mixture of *Bifidobacterium longum* and *Lactobacillus rhamnosus*. In another meta‐analysis involving 43 randomised control trials with 5531 IBS patients, *B. coagulans* exhibited the greatest efficacy in symptom relief, including alleviating abdominal pain and bloating. *L. plantarum* ranked first in improving QoL, and *L. acidophilus* had the lowest incidence of adverse events [112]. These findings suggest that probiotics, particularly strains like *L*. *acidophilus, B*. *coagulans* and *L*. *plantarum*, may improve IBS symptoms, QoL, abdominal pain and bloating. However, given the variability in patient responses, individualised probiotic treatments tailored to specific IBS subtypes and symptoms are crucial. Further research is needed to explore optimal strains, dosages and long‐term effects of probiotics in IBS treatment.

Prebiotics were first defined in 1995 as ‘non‐digestible food ingredients that beneficially affect the host by stimulating the growth and/or activity of one or a limited number of bacteria already resident in the colon’ [113]. More recently, they have been described as substrates selectively utilised by host microorganisms and conferring a health benefit [114]. Prebiotic soluble fibres are fermented by the intestinal microbiota into short‐chain fatty acids and enhance and stimulate the growth of resident bacteria in the colon [113, 115]. While multiple studies report an increase in *Bifidobacterium* abundance following prebiotic supplementation, evidence regarding their efficacy in alleviating IBS symptoms remains varied and inconclusive, as they may also be fermented and contribute to bloating or abdominal discomfort in hypersensitive patients. A systematic review and meta‐analysis of 11 randomised controlled trials with 729 patients by Wilson et al. [116] found that prebiotics had limited to no effect on IBS GI symptoms or improvement of QoL; however, they had also increased *Bifidobacteria*.

Differences in probiotic strains and formulations, as well as patient profiles, complicate generalisation of the intervention. Studies may also be limited by the self‐reported nature of symptom data. Additionally, baseline microbiota composition varies widely among patients, directly influencing treatment outcomes and highlighting the need for more robust clinical studies with standardised selection criteria and methods to optimise and personalise treatment.

## 6. Further Directions for Research and Limitations

### 6.1. Faecal Microbiota Transplantation (FMT) as a Potential Intervention

FMT has gained increasing attention as a promising alternative conservative treatment for a range of GI disorders. In the United States, the U.S. Food and Drug Administration permits the use of FMT under enforcement discretion for recurrent *Clostridioides difficile* infection (CDI) unresponsive to standard antibiotic therapy [117]. FMT has shown high success rates for CDI, reaching 94% [118, 119]. Recent research has also explored FMT as a potential treatment for IBS. FMT may directly target the low‐diversity gut microbiome associated with low‐grade inflammation and visceral pain. This transplantation involves transferring stool from a high‐diversity gut microbiome to a low‐diversity gut microbiome via colonoscopy, nasogastric or nasoduodenal tube or oral capsules. By introducing foreign microbiota, the gut microbiome of the recipient may increase in diversity, potentially restoring its influence over the GBA signalling pathways.

In a meta‐analysis by Abdelghafar et al. [120] comprised of eight randomised controlled trials with 472 patients, FMT had limited effectiveness in alleviating IBS symptoms regardless of the route of administration. While FMT improved QoL for some patients, its effects diminished over time, and the procedure was associated with an increased risk of adverse effects such as abdominal pain and constipation. Additionally, complexities in administering FMT for IBS include the lack of a definitive microbial signature associated with IBS and the absence of a universally defined healthy microbiome. These challenges arise from the intricate mechanisms of action by which FMT influences the microbiome, shaped by an array of factors such as donor and host microbiota composition and individual variations. These factors contribute to potential differences in the efficacy of FMT for alleviating IBS symptoms, further complicating its clinical applications [121].

While FMT has demonstrated success in animal models, its efficacy in IBS remains inconsistent due to varying donor‐ and host‐specific factors, as well as limitations in current knowledge. Standardisation and longitudinal studies would help to determine its sustainability. Donor microbiota composition influences the degree of clinical improvement, contributing to inconsistent outcomes. Host‐related factors, including baseline microbial composition and IBS subtype, also contribute to inconsistencies. While many studies examine changes in microbiota composition and symptom improvement post‐FMT, further research and observation, including its effects on the immune system, is required. Nevertheless, FMT offers promising insight into the therapeutic potential of directly addressing altered microbiota composition in IBS.

### 6.2. Limitations

Limitations of this literature review included determining the validity of the results presented across various studies due to different IBS subtypes and the highly individualised presentation of symptoms. Significant differences in the selection criteria, number of participants and the methods used also resulted in inconsistencies and potential biases, including information bias when self‐reporting symptoms and sampling bias that could misrepresent the wider population. Confounding factors influencing IBS pathogenesis, including age, sex and socioeconomic status, were also not accounted for in many studies. These aspects may have altered the extent of the reported associations between pathophysiological factors or interventions and IBS symptomology. Studies should strive to recruit substantial numbers of diverse participants diagnosed by the ROME IV diagnostic criteria to address these limitations. Potential confounders should be matched in those with and without IBS, and data should also be stratified based on varying degrees of confounding variables.

## 7. Conclusion

IBS is remarkably complex due to a vast array of factors contributing to GBA dysfunction, hypothesised to be the core of IBS pathophysiology. Such factors encompass genetic predisposition and physical and social environment—potential confounding factors that may contribute to varying results reported in studies. As the GBA involves bidirectional communication between the CNS and the ENS, the interconnections facilitating signalling within the network further add to its complexity, where dysfunction of the GBA is related to a cascade of effects influencing pain processing, gut motility, inflammation and GI‐related symptoms. While IBS may be diagnosed and classified into subtypes using the ROME IV diagnostic criteria, its cause and cure currently remain unknown.

Current evidence proposes stress management and dietary modification as the most suitable interventions to target the underlying pathophysiological mechanisms to manage GI‐ and non–GI‐related symptoms. Additionally, with further research and more trials, probiotic supplementation and FMT offer promising insights into a potential cure for IBS to address limitations in existing interventions.

With an increasing understanding and prevalence of IBS, there is a compelling need to develop more refined diagnostic criteria, as well as the identification of an IBS‐specific microbiome signature and diagnostic biomarkers. Research should also focus on directly targeting the underlying pathophysiological mechanisms. By considering all these elements, there is promising potential to develop a diverse array of sustainable treatments to improve the QoL of IBS patients.

## Ethics Statement

No ethical clearance required for this review article.

## Conflicts of Interest

The authors declare no conflicts of interest.

## Funding

The authors received no financial support for the research, authorship and/or publication of this article.
